# Linking phenological events in migratory passerines with a changing climate: 50 years in the Laurel Highlands of Pennsylvania

**DOI:** 10.1371/journal.pone.0174247

**Published:** 2017-04-12

**Authors:** Molly E. McDermott, Lucas W. DeGroote

**Affiliations:** Powdermill Nature Reserve, Carnegie Museum of Natural History, Rector, Pennsylvania, United States of America; Utrecht University, NETHERLANDS

## Abstract

Advanced timing of both seasonal migration and reproduction in birds has been strongly associated with a warming climate for many bird species. Phenological responses to climate linking these stages may ultimately impact fitness. We analyzed five decades of banding data from 17 migratory bird species to investigate 1) how spring arrival related to timing of breeding, 2) if the interval between arrival and breeding has changed with increasing spring temperatures, and 3) whether arrival timing or breeding timing best predicted local productivity. Four of 17 species, all mid- to long-distance migrants, hatched young earlier in years when migrants arrived earlier to the breeding grounds (~1:1 day advancement). The interval between arrival on breeding grounds and appearance of juveniles shortened with warmer spring temperatures for 12 species (1–6 days for every 1°C increase) and over time for seven species (1–8 days per decade), suggesting that some migratory passerines adapt to climate change by laying more quickly after arrival or reducing the time from laying to fledging. We found more support for the former, that the rate of reproductive advancement was higher than that for arrival in warm years. Timing of spring arrival and breeding were both poor predictors of avian productivity for most migrants analyzed. Nevertheless, we found evidence that fitness benefits may occur from shifts to earlier spring arrival for the multi-brooded Song Sparrow. Our results uniquely demonstrate that co-occurring avian species are phenologically plastic in their response to climate change on their breeding grounds. If migrants continue to show a weaker response to temperatures during migration than breeding, and the window between arrival and optimal breeding shortens further, biological constraints to plasticity may limit the ability of species to adapt successfully to future warming.

## Introduction

Migratory birds living in seasonal environments, such as temperate forests where food supplies are restricted temporally, must optimize migration and reproductive timing to reduce fitness costs. Global climate is changing at an unprecedented rate [1], presenting a novel challenge for migrants by inducing phenological shifts in food availability. As such, individual birds must demonstrate plasticity to adapt to climate change in order to avoid negative fitness and survival repercussions. Fortunately, high plasticity in spring migration and reproductive timing [2–5] among songbirds has allowed many species to maximize productivity in the face of yearly climate variation.

Breeding phenology is highly variable interannually for bird populations and is determined by the interaction between photoperiod and environmental signals such as temperature, leaf emergence, and food abundance [6–9]. Early arrival during spring migration [10–15] and early laying dates [16–22] have resulted from warming temperatures. Early breeding may result from early arrival when emergence of vegetative and invertebrate resources advance concurrently [23–27], thereby leading to earlier hatching and fledging dates [21,28]. Breeding is constrained by arrival timing for migratory birds; thus, we expect a strong linkage between migratory and breeding phenology [29,30].

Timing of migration and breeding ultimately influences fitness in a variety of ways [31] and can carry over into the following breeding season [32]. Although the earliest birds may face inhospitable conditions, severe weather, and low food resources, individuals arriving late may fail to acquire a mate or miss peak food availability during migration or breeding [33–36]. In fact, early arriving birds tend to occupy better territories, have higher quality mates, and raise more broods [16,34,37,38]. For example, individual American Redstarts (*Setophaga ruticilla*) that arrived early hatched nestlings with a greater mass and sooner than later arriving individuals [35]. Early breeding can also result in higher reproductive success as was found for populations of Song Sparrows (*Melospiza melodia*; [39]) and Pied Flycatchers (*Ficedula hypoleuca*; [40]). Thus, increased reproductive success is possible if mismatches in timing of peak resource abundance do not occur [41].

There is ample evidence that many bird species are modifying migratory timing and subsequent breeding in response to climate change with individuals responding to annual climate variation (above). Despite this evidence, rigorous examination of the link between long-term migratory and breeding phenology is lacking. Furthermore, the fitness consequences migratory and breeding period variation have yet to be analyzed with a long-term, multi-species dataset. In this study, we used five decades of banding data from a constant effort mist-netting station in western Pennsylvania to investigate these linkages with climate change and productivity for 17 migratory bird species. Our dataset is uniquely consistent and precise, having been collected year round in a standardized effort across 53 years by only a few highly skilled bird banders. Specifically, we aim to address three main questions regarding shifts in breeding phenology. First, does earlier arrival lead to earlier breeding? Second, is the interval between arrival on breeding grounds and breeding shorter when spring temperatures are warmer; i.e., is either phenological event more sensitive to warming? We explored rates of change using time interval metrics between adult spring arrival date, breeding initiation, and juvenile appearance. As a subquestion, we investigated whether breeding advancement resulted from earlier nesting initiation and/or reduced nesting periods using a subset of the data. Third, focusing on population-level productivity, we asked whether arrival timing or breeding timing best predict reproductive success for 17 migratory bird species.

## Materials and methods

### Bird data

Bird banding data were collected from 1961–2014 at Powdermill Nature Reserve (PNR; 400 masl, 40°10’N, 79°16’W) in the Laurel Highlands of Westmoreland Co., PA, USA. The 10-ha banding area is comprised of scrub, wetland, and old field habitats. The station is operated year-round, typically 3–5 days per week during the breeding (June– 15 August) season and 6 days per week during spring migration (April—May). Number of mist-nets (20–60) and number of hours opened (~6 h) were recorded daily. Captured birds were fitted with individually numbered USGS aluminum leg bands. For each individual captured we recorded age, sex, wing length, fat score, presence of brood patch, and presence of gravidity prior to release.

Three timing metrics were derived from capture data using Julian date. We calculated spring arrival date as the 10% quantile of captures each year during spring migration. June 7 was used as a cut off for the terminus of spring migration because passage migrants have departed the study area by that date (11). Observations prior to March 1 were removed to eliminate birds that may have overwintered at PNR. We included between-year recaptures, but each individual was only used once per year in the 10% calculation (first capture date of the season). Migration arrival dates represent mostly passage migrants. Thus, we made the assumption that local breeders’ arrival was directly related to passage migrant arrival as a whole.

Secondly, we calculated juvenile appearance date to quantify reproductive timing. To determine breeding seasonal cut-off dates for reproductive data, capture frequencies by Julian day for each species were examined in addition to records on timing of reproductive events from the Second Pennsylvania Breeding Bird Atlas [42]. We used conservative end of breeding season dates to eliminate most autumn migrants but still include locally hatched birds and dispersers. Juvenile appearance date was estimated yearly for each species at which point 10% of juveniles had been captured during the breeding season within these pre-defined windows. Juveniles were only counted once, recaptures were omitted from the dataset. Years with <10 captures of juveniles were dropped, and species were only analyzed if >10 juveniles were captured for at least 10 years.

Lastly, for a subset of species we were able to calculate a surrogate for breeding initiation, a second indicator of reproductive timing. We determined the Julian date for each species at which point 10% of the breeding females (gravid or with brood patch) were captured by year. Only definitive brood patch scores were used; i.e., stages from the initiation (defeathering) of brood patch, complete vascularization, and subsequent patch refeathering [43]. The stages of brood patch and egg development, hormonally controlled, are tightly linked to breeding and thus represent excellent indicators of breeding phenology. For this breeding condition metric, we used species having >10 years of data with >10 recorded individuals with presence of breeding condition per season.

The 10% quantile was used in all metrics to reduce the effects of outliers. Temporal distributions such as quantiles are preferable to distributional extremes (e.g., first appearance/arrival) for reflecting phenology of populations, especially in cases of low sample size and imperfect detectability [12,44].

An index of breeding productivity was calculated on a yearly basis as (juvenile capture rate)/(adult capture rate) within the breeding window for all 17 migratory species (Table 1). Number of captures per 100 mist-net hours (capture rate) was determined for each species, age class, and year. Although not comparable among species due to interspecific differences in capture probability, this index has been useful to track changes in productivity [45–47].

**Table 1:** Species specific first day of 3-week period used for climate variables based on greatest correlation to each of three response variables. Spring Temp = mean temperature at breeding initiation. Only dates for variables included in analyses are presented. The three response variables are the interval in days between spring arrival and juvenile appearance (10% quantile for both; "Arrival-Juvenile Appearance" the interval between arrival and breeding initiation as indicated by female breeding condition ("Arrival-Breeding"), and the interval between breeding initiation and juvenile appearance ("Breeding-Juvenile Appearance").

Species		Mig_a_	Hab_b_	Broods_c_	Arrival-Juvenile Appearance Spring Temp	Arrival-Breeding Spring Temp	Breeding-Juvenile Appearance Spring Temp
Ruby-throated Hummingbird	*Archilochus colubris*	long	forest	2	17-May		
Eastern Phoebe	*Sayornis phoebe*	short	open	2	5-Apr		
Red-eyed Vireo	*Vireo olivaceus*	long	forest	2	16-Apr	16-Apr	15-May
House Wren	*Troglodytes aedon*	short	open	2	24-Apr	9-Apr	28-Apr
Wood Thrush	*Hylocichla mustelina*	long	forest	2	27-Apr	26-Apr	4-Apr
American Robin	*Turdus migratorius*	short	open	3	31-Mar		
Gray Catbird	*Dumetella carolinensis*	long	open	3	25-Apr	24-Mar	24-Mar
Cedar Waxwing	*Bombycilla cedrorum*	short	forest	2	28-Apr	28-Apr	28-Apr
Ovenbird	*Seiurus aurocapilla*	long	forest	2	7-May		
Common Yellowthroat	*Geothlypis trichas*	long	open	2	28-May	2-May	2-May
Hooded Warbler	*Setophaga citrina*	long	forest	2	30-Apr		
American Redstart	*Setophaga ruticilla*	long	forest	1	1-Apr	1-Apr	15-Apr
Yellow Warbler	*Setophaga petechia*	long	open	1	12-Apr	6-May	16-Apr
Field Sparrow	*Spizella pusilla*	short	open	2	26-Apr	9-Mar	
Song Sparrow	*Melospiza melodia*	short	open	3	2-Apr	4-Apr	29-Mar
Indigo Bunting	*Passerina cyanea*	long	open	2	8-May	1-May	
American Goldfinch	*Carduelis tristis*	short	open	1	7-Jun	27-Apr	27-Apr

^a^ General migration distance

^b^ Breeding habitat guild. Open = generalist and early-successional. Forest = mid to late-successional forest breeders

^c^ Number of broods that are typically raised in a breeding season for the study area

### Climate data

Data were accumulated in three-week intervals from February-August for the study period from weather stations (N = 21) within 40 km of PNR to account for climatic conditions near the breeding grounds. We retrieved average daily temperature from the National Oceanic and Atmospheric Administration (NOAA) climate database (http://www.ncdc.noaa.gov/cdo-web/search). Temperature was averaged over all possible three-week intervals. We decided *a priori* to restrict our analysis to three-week temperature intervals because prior research has demonstrated that migrants can adjust their migration and nesting phenology in response to short-term (i.e. two to four week windows) proximate climatic cues [11, 20, 30, 48, 49].

We used a sliding window approach (e.g., [20,21,30,48,49]) to identify the three week time period for which our climate variable (mean spring temperature) and response variable (below) were most closely correlated for each species (Table 1). Spring climate variables were chosen from months preceding and at the initiation of breeding season for each species studied, when they would be most influenced by resource availability due to plant and arthropod phenology.

### Data analyses

First, we compared timing of spring arrival (10% quantile) to juvenile capture date (10% quantile) for 17 species (Table 1) using generalized linear regression models and estimated best fit lines to describe the relationship between these two variables.

Next, we modeled the effects of spring temperature and year (predictors) on time interval between arrival and juvenile appearance (response variable) using linear regression. Seventeen migratory species met the requirements for this analysis of the arrival-juvenile appearance interval, derived from our 10% capture timing metrics. This interval was defined as the difference between arrival on breeding grounds and appearance of young (juvenile appearance date—arrival date). To further investigate the potential mechanisms behind changes in phenology, we regressed two additional time intervals on spring temperature and year. A subset of years with adequate data on female breeding condition was used to split the time between the spring arrival of adults and appearance of juveniles into two distinct intervals: time interval between arrival and breeding initiation (10% female breeding condition date), and time interval between breeding initiation and appearance of juveniles.

To assess possible causal factors influencing fitness, we used an information-theoretic approach to examine whether arrival time or reproductive timing best explained yearly productivity index for 17 species [50]. The decision to include arrival time and breeding time in separate models was based on results from the first analysis. The candidate model sets included the following: 1.) a null model (no effects), 2.) a model with spring arrival timing, 3.) a model with juvenile appearance timing, and 4.) a global model which included both the timing of spring arrival and juvenile appearance. We selected the most parsimonious model using AIC adjusted for small sample sizes (AIC_*c*_), ranked according to the top model as ΔAIC_*c*_, and we evaluated models based on Akaike weight. Models with a ΔAIC_*c*_ < 2 were considered equally plausible [50]. All statistical analyses were performed using R version 3.0.3 [51]. Model-averaged parameter estimates were obtained for each candidate set (weighted averages of all competing models) using the ‘AICcmodavg’ package [52]. This package computes unconditional standard errors and 95% confidence intervals, which we used to evaluate relationships between variables.

### Ethics statement

This research involved mist-netting and banding passerines, but no experiments on or collection of birds. Procedures were followed in accordance with state and federal legislation. This research was conducted using ethical guidelines following the North American Banding Council code of ethics (http://www.nabanding.net/banders-code-of-ethics/) and the Ornithological Council's guidelines to the use of wild birds in research (http://naturalhistory.si.edu/BIRDNET/documents/guidlines/Guidelines_August2010.pdf). We conducted mist-netting and banding under a Pennsylvania state permit and federal (U.S. Geological Survey) permit number 08231.

## Results

### Relationship between spring arrival and timing of breeding

**Fig 1:**
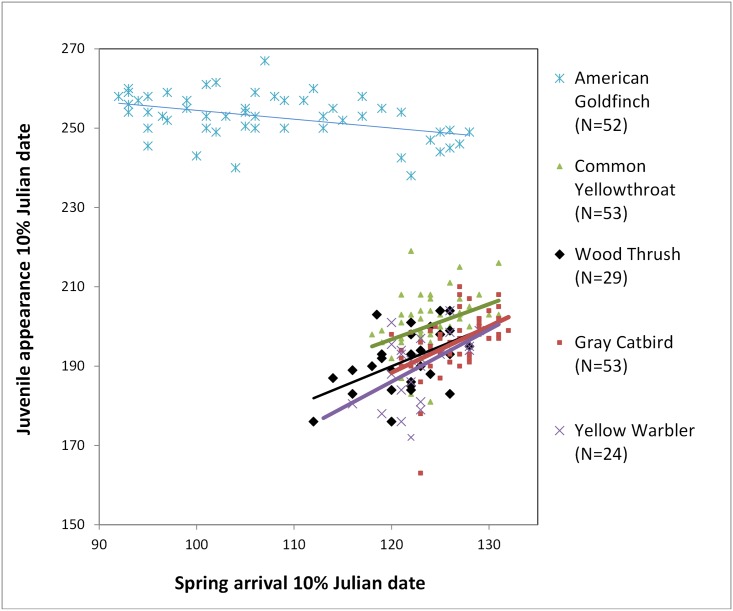
Seasonal timing of juvenile appearance (day at which 10% of young were captured) as related to spring arrival (day at which 10% of spring adult migrants were captured). Bird data were collected from 1961–2014 at a constant effort mist-netting station in Pennsylvania.

Most species showed no significant linear pattern (12 species), four species of mid to long-distance migrants hatched young earlier in years when migrants arrived earlier to the breeding grounds: Wood Thrush (scientific names in Table 1; *F*_1,27_ = 9.38, *P* = 0.005), Gray Catbird (*F*_1,51_ = 15.06, *P*<0.001), Common Yellowthroat (*F*_1,51_ = 8.88, *P* = 0.004), and Yellow Warbler (*F*_1,22_ = 7.33, *P* = 0.013). The relationship ranged from 0.9 days (Common Yellowthroat) to 1.3 days (Yellow Warbler) advancement in appearance of young to each day of advancing spring arrival (Fig 1). One species, the late-breeding American Goldfinch, showed a negative relationship (*F*_1,50_ = 10.73, *P* = 0.002; Fig 1), exhibiting later appearance of young in years of earlier arrival (Fig 1).

### Timing between phenological events changing with spring temperatures

The time interval between spring arrival on breeding grounds and appearance of juveniles decreased with warmer spring temperatures for 71% of migratory species analyzed (12 of 17 species; Table 2), and was evident for both single and multiple brooders (Table 1). The interval was reduced by 1–6 days for every 1°C increase in spring temperature. Seven species also reduced the interval between spring arrival and juvenile appearance over time (1–8 days per decade); despite a nonsignificant increase in spring temperatures over the study period in this region of Pennsylvania [28]. Conversely, American Goldfinches showed increasing intervals across years analyzed (4 days increase per decade; Table 2). The decrease in lag time between spring arrival and juvenile appearance for the majority of species analyzed met our *a priori* expectations and led us to examine changes within this interval.

**Table 2:** The relationship of three time interval variables with spring temperature (Table 1) and year effects. Arrival-Juvenile = the interval between spring arrival and juvenile appearance (10% quantiles). Arrival-Breeding = the interval between spring arrival and breeding initiation as indicated by presence of female breeding condition (10% quantiles). Breeding-Juvenile = the interval between breeding initiation and juvenile appearance date. Parameter estimates for slopes are listed for significant effects (§ = P<0.10, * = P<0.05, ** = P<0.01, *** = P<0.001). N = years with >10 captures. Temp = days per 1°C increase.

Species	Arrival-Juvenile Appearance			Arrival-Breeding			Breeding-Juvenile Appearance			Arrival $\bar{x}$ capt_a_	Breeding $\bar{x}$ capt_b_	Juvenile $\bar{x}$ capt_c_
	*N*	Temp	Year	*N*	Temp	Year	*N*	Temp	Year			
Ruby-throated Hummingbird	45	*-1.23	NS							61.8		39.4
Eastern Phoebe	36	NS	NS							15.2		38.5
Red-eyed Vireo	49	*-1.28	NS	24	§ -3.89	§ 0.68	23	NS	NS	71.8	29.6	36.7
House Wren	44	**-4.10	NS	14	NS	NS	12	*8.40	NS	24	12.7	20.7
Wood Thrush	29	** -1.99	**-0.29	13	*-3.29	NS	13	***-3.60	NS	25.5	14.6	25.4
American Robin	27	NS	*-0.75							23.3		19.7
Gray Catbird	53	NS	*-0.12	25	*-2.03	NS	25	NS	NS	118.1	31.7	90.5
Cedar Waxwing	50	*-1.72	**-0.27	34	*-3.67	NS	33	NS	NS	103.7	36.5	105.3
Ovenbird	20	**-3.42	NS							18.7		22.2
Common Yellowthroat	53	* -1.64	**-0.17	30	*-3.02	NS	30	*2.84	NS	92.8	16.3	56.8
Hooded Warbler	31	*** -1.69	**-0.25							28.9		46.3
American Redstart	41	§ -1.35	NS	21	**-2.42	NS	22	*-2.10	NS	31.8	21.8	59
Yellow Warbler	24	NS	**-0.40	16	NS	NS	10	NS	NS	53.2	13.9	16.1
Field Sparrow	22	*-2.00	NS							74.3		40.9
Song Sparrow	53	*-2.06	NS	20	NS	NS	20	NS	NS	199.1	21.4	84.9
Indigo Bunting	38	* -1.93	NS	11	**-7.70	NS				43.2	12.2	21.8
American Goldfinch	52	*-2.70	***0.46	41	NS	NS	40	NS	NS	189.1	28.3	56.9

^a^ Mean captures per year used for timing of spring arrival in years with >10 captures.

^b^ Mean captures per year used for timing of breeding initiation in years with >10 captures.

^c^ Mean captures per year used for timing of juvenile appearance in years with >10 captures.

In warmer springs the interval between arrival and breeding shortened for six species (55%) and lengthened for none of the 11 species analyzed (Table 2). Year was nearly significant for one species; Red-eyed Vireos showed a longer delay in onset of breeding over time based on female breeding condition.

Of the of the 10 migrant species analyzed, the interval between breeding initiation and appearance of juveniles in warmer springs shortened for two species (Wood Thrush and American Redstart) while this interval lengthened for two species (House Wren and Common Yellowthroat; Table 2). No species showed a significant year effect.

**Fig 2:**
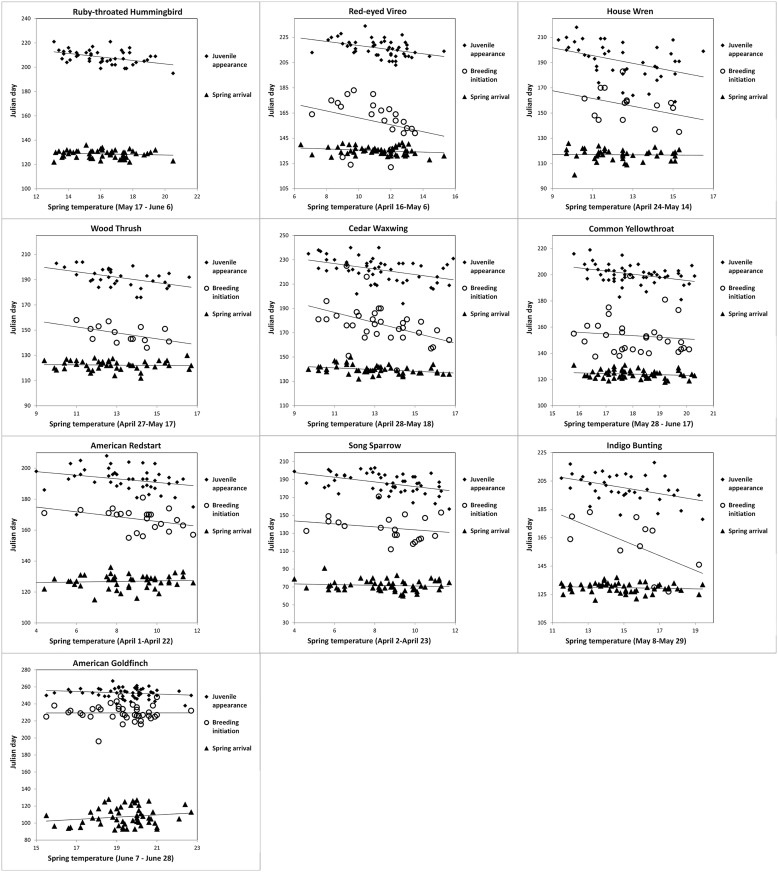
Phenology comparison for three events as a function of spring temperature for 10 migratory bird species. The events are timing of juvenile appearance (Julian day at which 10% of young were captured), breeding initiation (Julian day at which 10% of females in breeding condition were captured), and spring arrival (Julian day at which 10% of spring adult migrants were captured). Mean spring temperature for each species was calculated from the three-week time period most correlated with the time interval between spring arrival and juvenile appearance each year (Table 1). Bird data were collected from 1961–2014 at a constant effort mist-netting station in Pennsylvania.

In general, the magnitude of change as a function of spring temperature was greater for these two within-period intervals (arrival-breeding and breeding-juvenile appearance) than the entire arrival to juvenile appearance period (Table 2). In most species the shortening of the overall interval with increasing temperatures can be attributed to advancing appearance of juveniles accompanied by a fairly steady or slightly advancing arrival on the breeding grounds (Fig 2). From our analyses there is evidence that rates of breeding initiation and juvenile appearance are changing similarly with temperature (i.e., comparable slopes), implying that onset of breeding occurs more quickly in warmer springs at the population level for many species we studied (Fig 2).

### Effects of phenology on productivity

**Fig 3:**
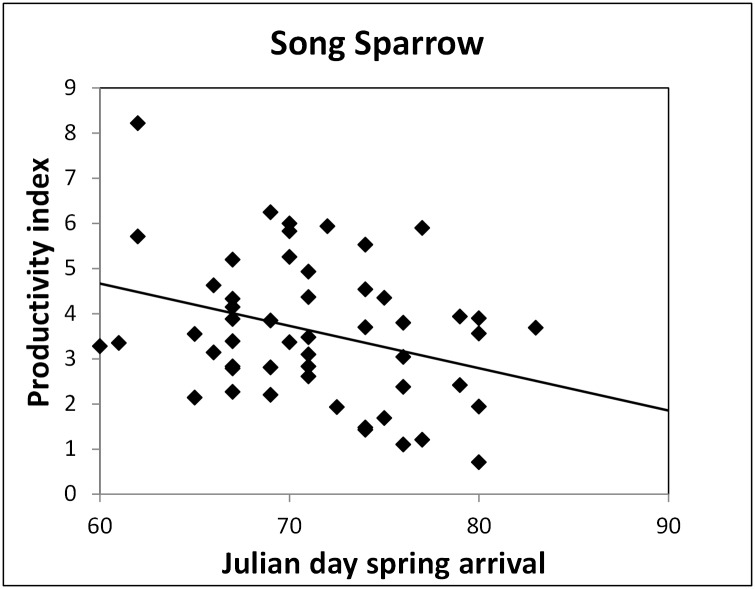
Productivity index, (juvenile capture rate)/(adult capture rate), as a function of adult spring arrival date (Julian day at which 10% of spring adult migrants were captured) for the Song Sparrow (N = 53 years). This is the only species showing a significant increase in productivity with earlier arrival.

Timing of adult spring arrival and appearance of young were in general poor predictors of local bird productivity (Table 3). Timing of juvenile appearance was only associated with productivity for Hooded Warblers and American Redstarts; the parameter estimate was nonsignificant in all other species where it appeared in top models (Table 3). Later juvenile appearance was associated with higher productivity (capture rate of young) for both species. The slope for spring arrival timing was significant for two species for which it was in top weighted models (ΔAIC_*c*_ < 2). Of these, Song Sparrows produced more young in years with early arrival (Table 3; Fig 3), while Common Yellowthroats produced fewer young in years of early arrival.

**Table 3:** Candidate model weights (model probabilities) comparing 4 models for productivity index, when ΔAICc <2. Model-averaged parameter estimates and unconditional standard errors (SE) are presented except when the 95% confidence interval overlapped zero (termed NS = non-significant). N = years with >10 captures.

Species	*N*	Candidate Model Akaike weights				Parameter estimates (SE)	
		Null	SA	JA	Global	Spring arrival	Juvenile appearance
Ruby-throated Hummingbird	45	0.25	0.48	.	.	NS	NS
Eastern Phoebe	30	0.39	0.19	0.31	.	NS	NS
Red-eyed Vireo	49	0.51	0.19	0.21	.	NS	NS
House Wren	36	0.52	.	0.22	.	NS	NS
Wood Thrush	29	0.56	.	.	.	NS	NS
American Robin	27	0.56	0.22	.	.	NS	NS
Gray Catbird	53	0.5	0.21	0.22	.	NS	NS
Cedar Waxwing	50	0.31	.	0.44	.	NS	NS
Ovenbird	20	0.29	0.34	0.16	0.21	NS	NS
Common Yellowthroat	50	.	0.52	.	0.22	0.38 (0.18)	NS
Hooded Warbler	31	.	.	0.59	.	NS	0.23 (0.10)
American Redstart	41	.	.	0.63	.	NS	0.14 (0.06)
Yellow Warbler	24	0.38	.	0.41	.	NS	NS
Field Sparrow	22	0.42	.	0.39	.	NS	NS
Song Sparrow	53	.	0.60	.	0.33	-0.09 (0.03)	NS
Indigo Bunting	38	0.44	.	0.30	.	NS	NS
American Goldfinch	52	0.27	0.26	0.32	0.15	NS	NS

SA = Model with spring arrival date (10% quantile). JA = Model with juvenile appearance date (10% quantile).

## Discussion

Dissecting components of phenology allows us to better understand the relative importance of climate and carry-over effects of timing on reproductive performance. Individual phases within the avian lifecycle might be accelerated either due to a linkage with warmer temperatures or because preceding phenology was accelerated. For example, a species might respond to warmer temperatures by arriving earlier, but delay breeding a fixed number of days, and earlier fledging would entirely result from early arrival. Our results disentangle the interaction between climate, phenology and fitness for various species, and show that not all species respond to climatic shifts equally.

Arrival timing had little effect on timing of juvenile appearance, yet for most species, juveniles appeared earlier at PNR in warm springs (Fig 2). The earlier appearance of young may have resulted from either early onset of breeding or a shortened time interval between nesting to fledging. Warmer temperatures appeared to drive advanced onset of breeding for seven of the 17 species (Ruby-throated Hummingbird, Red-eyed Vireo, Wood Thrush, Gray Catbird, Cedar Waxwing, Common Yellowthroat, Indigo Bunting; Table 2). Only four species showed a link between early arrival and early breeding; Wood Thrushes, Gray Catbirds, Common Yellowthroats, and Yellow Warblers all advanced breeding by one day for every one day of earlier arrival. Changes in resource abundance along migratory routes may allow these mid-distance migrants to arrive earlier in warm springs, despite not having the same climate cues on their wintering grounds [53].

However, 12 of 17 species showed no relationship between early arrival and early breeding onset within a given year, consistent with the results of [54]. Incubation periods were shorter in warmer years in only two species (Breeding-Juvenile in Table 2; Wood Thrush and American Redstart). Seven species also showed advancing juvenile appearance across years, indicating some other force is driving early appearance of young that is neither strictly related to temperature nor arrival timing (1–8 days per decade: Wood Thrush, American Robin, Gray Catbird, Cedar Waxwing, Common Yellowthroat, Hooded Warbler, and Yellow Warbler). One prominent exception to the trend of early breeding lies with the American Goldfinch, a late summer breeder. We found the interval between arrival and juvenile appearance shortened in warmer springs with delayed arrival resulting in later breeding over time (see [28], for more detail).

The differences between the aforementioned species suggest that plasticity in breeding biology is partitioned and limited. Other studies showing plasticity in arrival and breeding phenology [2–5] have not dissected phenological components to the degree we have done here. For example, the decrease in time from arrival to juvenile appearance with rising temperature is significant in 12 of 17 species. Partitioning the period is informative: for arrival to breeding, seven of 14 species were significant, and for the breeding to juvenile appearance interval only two of 12 were significant. However, when advancement was significant within the partitions, the magnitude (i.e., number of days/°C) is greater than for the whole period considered together. Thus, even though both onset of breeding and time to fledging may contribute to a species’ phenological response, there is a tighter linkage with temperature and an individual component (either onset of breeding or time to fledging). The fact that 12 of 17 species here show no relationship between early arrival *per se* and early breeding onset implies that these species, when they arrive early, are not simply following a schedule, but apparently are responding to other climatic or environmental variables.

One remaining question is what information is available to long distance migrants that might accelerate their arrival time? It stands to reason that long-distance migrants are more likely to arrive on breeding grounds to find that the vegetative phenology is more advanced, with the migrants effectively arriving “late.” In hardwood forests such as those found at our site, the flexibility of many migratory species buffers them from resource timing mismatches; many long-distance migrants have a diverse prey base that is seasonally stable and have exhibited plastic responses in reproductive timing [41,55]. Breeding could advance at a greater rate than migration because breeding phenology is more sensitive to temperature for long-distance migrants [56]. We found that warmer temperature appears to drive advanced onset of breeding for seven species. (Red-eyed Vireo, Wood Thrush, Gray Catbird, Cedar Waxwing, Common Yellowthroat, American Redstart, Indigo Bunting, Table 2). Even short-distance migrants like House Wren and Cedar Waxwing bred much earlier in warmer springs in absence of strong shifts in arrival timing (Fig 2). This might suggest a linkage with earlier timing of spring leaf expansion, food abundance, or proximate cues such as temperature [8,9,20,27,57–61]. Thus, these migrants may be adjusting the onset of breeding based on plant phenology, which has shifted with temperature at a rate three times that of spring arrival timing [11], similar to the rate of reproductive advancement for many species [28].

Birds can modify the interval between laying and hatching by adjusting clutch size, varying onset of incubation, increasing the intensity of incubation to accelerate hatching, and investing differentially on eggs [62,63]. Total incubation time can be decreased to an extent under favorable conditions, but there are biological limits to the speed in which eggs hatch and nestlings fledge. Fledging times may be reduced in warmer years via an abundance of resources or reduced incubation costs [21,64–67]. Yet, we found little evidence for these effects. For long-distance migrants especially, it will not be sustainable to advance breeding faster than arrival with long-term warming. With a minimum 2–3.5°C increase in temperature predicted by the end of the current century (1), we expect the migratory species we studied to further advance arrival by >2–7 days [11] and breeding by >2–10 days [28]. In extremely warm springs (mean April/May temperatures of 15–20°C; Fig 2), some species may arrive too late to take advantage of optimal timing. For example, Red-eyed Vireos, Indigo Buntings, Cedar Waxwings and Wood Thrush might only need a 5°C increase before mismatches occur (Fig 2). Furthermore, despite strong patterns of advanced timing of breeding accompanying warmer seasons, there is no apparent fitness advantage to most species in our study. Of 17 species studied, only the Song Sparrow shows any increase in fitness associated with accelerated arrival (Fig 3, and below), indicating that the disruption of historical phenological patterns carries more risk than reward.

### Effects of phenology on productivity

We found little evidence that timing of either arrival on breeding grounds or timing of breeding contributed to increased productivity at the population level. However, there was evidence that more young may be produced given shifts to earlier spring arrival for the multi-brooded Song Sparrow. Contrarily, Common Yellowthroats were more productive with later arrival, and American Redstarts and Hooded Warblers were more productive in years when juveniles appeared later.

Many studies have found that early arrival and breeding result in increased reproductive success in passerines when food availability allows, e.g., by raising multiple broods [55,68–71] or larger clutches [23,35,72,73]. Early arrival on breeding grounds has been associated with increased fledged young [40,74] including in Song Sparrows as we found [39] resulting in directional selection towards early arrival [27,35,36,75–78].

On the other hand, productivity or reproductive success may not be affected by timing of arrival or laying, as 13 of 17 species we studied demonstrated. Advanced laying dates may not impact productivity (i.e., a neutral effect; [79]), and some species lay smaller clutches earlier in the season [80]. If mismatches with timing of peak resource abundance increase with warming, it may be less effective to raise a second brood [81]. Other fitness components, such as nest success of individual pairs and adult survival (e.g., [82]) may be impacted by phenological shifts. Consequently, we hesitate to say that shifts in timing will not have fitness impacts in the species we studied. Rather, our results suggest that shifts in timing may have little effect on local productivity either because advancing phenology does not convey improved fitness or because our local breeding populations may track resource phenology by necessity to avoid mismatches and maintain consistent levels of productivity. In fact, changes in phenology were related to changes in productivity for only 24% of the species we studied, while prior research in the same study system demonstrated that temperature and precipitation explained changes in productivity for 57% and 75% of the species studied, respectively [28]. Given that nearly 3 out of 4 migratory birds in our study area advanced or delayed the window between arrival and breeding, changes in climate appear to be ultimate drivers of phenological shifts and productivity.

## Conclusions

This study provides novel insight into linkages between phenological events in migratory birds uniquely capitalizing on five decades of bird banding data from a long-term monitoring program. We observed considerable phenological plasticity in most species studied, finding that even the farthest migrating species reproduced earlier in warm springs. Arrival timing, however, had no effect on breeding phenology or productivity for most species at PNR. Instead, breeding phenology advanced 1 to 6 days more quickly than arrival timing per 1°C increase, indicating that migratory birds are likely tracking resource phenology upon arrival. If migrants persistently show a weaker response to temperatures during migration than breeding, and the window between arrival and optimal breeding continues to shorten, biological constraints to plasticity may limit the ability of species to adapt successfully in the long run.
